# Abstracts of Scottish Vision Group 2023 Meeting

**DOI:** 10.3390/vision8010012

**Published:** 2024-03-11

**Authors:** Paul George Lovell, Rebecca J. Sharman

**Affiliations:** Division of Psychology and Forensic Sciences, School of Applied Sciences, Abertay University, Dundee DD1 1HG, UK; r.sharman@abertay.ac.uk

**Keywords:** camouflage, visual cognition, binocular coding, crowding, visual foraging, visual search, visual discomfort, football

## Abstract

Scottish Vision Group has become a yearly event for vision scientists based in Scotland since its inception in 2001. Each year, the conference is hosted at a different location and organised by a different team. The 2023 SVG meeting was hosted in the city of Dundee by Abertay University. Delegates travelled from the United Kingdom, Europe and beyond. The meeting started with a roundtable panel discussion sponsored by Meta Reality Labs. The roundtable, titled The Past, Present and Future of Visual Search, was organised and presented by Árni Kristjánsson (Iceland), Ioan Smart (Abertay) and Ian Thornton (Malta). The MDPI Keynote lecture was introduced by Professor Andrew Parker (Oxford University and Otto-von-Guericke University in Magdeburg, Germany) and presented by Prof. Timothy Ledgeway (Nottingham) on sensory eye dominance and plasticity in adult binocular vision. The remaining two days of the conference hosted a wide range of talks on topics ranging from insect navigation to visual illusions, facial recognition and binocular coding. The Saturday evening saw a special event where delegates explored the sensory properties of a range of single-malt whiskies. Here, we present a selection of abstracts for the various talks and posters.

## 1. META Round Table Discussion—Visual Search: Past, Present & Future

Ioan Smart ^1^, Árni Kristjánsson ^2^ and Ian Thornton ^3^
^1^ Division of Psychology, School of Applied Sciences, Abertay University, UK
^2^ Faculty of Psychology, University of Iceland
^3^ Department of Cognitive Science, Faculty of Media & Knowledge Sciences, University of Malta, Msida, Malta

**Abstract:** Broadly speaking, we use visual search paradigms as an analogy for how one interacts with the world. Classic visual search paradigms have proven useful in our understanding of vision, yet these paradigms have been steadily advancing in the direction of ecological validity. Namely, we have seen a shift from single target paradigms to multiple target paradigms and a shift from 2-dimensionality to 3-dimensionality, particularly via the use of desktop and immersive VR technologies. With this comes concern over the existing search template model; how can the concept of a “search image” account for the variability of objects encountered in the world? How do changes in temporal scale affect search when moving from simplified static to more complex, dynamic experimental scenarios? For this round table talk we will discuss: (i) the history of visual search; (ii) how the addition of factors that make visual search more realistic impact the visual search paradigm; and (iii) how we see visual search research going forward.

## 2. MDPI Keynote Talk: Sensory Eye Dominance and Plasticity in Adult Binocular Vision

Tim Ledgeway
University of Nottingham

**Abstract:** It has long been believed that the visual system is relatively hard-wired after the closure of a “critical period”. However, recent research, using the technique of short-term monocular deprivation, has found that the balance of the two eyes in adult humans can be altered by introducing short-term modifications to visual input in one eye. For example, depriving one eye of spatial information for a few hours can shift sensory dominance, measured with a binocular rivalry task, in favour of the previously deprived eye. However, the mechanism underlying this phenomenon is unclear. In this talk I will review examples of our recent research that have begun to shed light on the nature of binocular interactions and plasticity in the healthy human visual system. I will explore the patterns of individual differences in sensory eye dominance in healthy adults, show that sensory eye dominance is not simply predicted by differences in monocular visual function and demonstrate that both low-level sensory input and higher-level processes can shape sensory eye dominance. Taken together these studies suggest a rich and complex pattern of inter-ocular interactions in adult binocular vision, that may also have important implications for rebalancing the two eyes in clinical disorders of binocular vision.

## 3. The Visual Cortex in Migraine with Aura Has More Neural Noise

Louise O’Hare ^1^, Paul B. Hibbard ^2^ and Arnold J. Wilkins ^2^
^1^ School of Social Sciences, Nottingham Trent University
^2^ Department of Psychology, University of Essex

**Abstract:** Migraine is a common neurological disorder with strong associations to visual perception in terms of hallucinations prior to attacks, aversion to light during the attack, and differences in visual perception in between attacks. Individuals with migraine aura show increased neural responses, but typically poorer performance on visual tasks. Increased neural noise has been suggested as a possible reason for this apparent paradox, however the mechanisms for this have not yet been directly investigated. We measured the steady-state visual evoked potential for a 3-degree diameter sine grating that increased in contrast and faded to mid-grey in a sine-wave temporal profile (flickering) at 5 and 17 Hz in 15 individuals with migraine aura and 15 healthy controls. The electrophysiological response of the migraine group showed a lower overall signal-to-noise ratio, that is the response to the flickering stimulus was less pronounced compared to the background EEG activity, providing electrophysiological evidence that the migraine brain is “noisier” compared to controls.

## 4. Dynamic Resource Allocation in Spatial Working Memory during Full and Partial Report Tasks

Siobhan M. McAteer, Anthony McGregor and Daniel T. Smith
Department of Psychology, Durham University

**Abstract:** Serial position effects are well documented in working memory literature. Studies of spatial short-term memory that rely on binary response, full report tasks tend to report stronger primacy than recency effects. In contrast, studies that utilize a continuous response, partial report task report stronger recency than primacy effects (Gorgoraptis et al., 2011). This study explored the idea that probing spatial working memory using full and partial continuous response tasks would produce different distributions of visuospatial working memory resources across spatial sequences, and therefore explain the conflicting results in the literature. Experiment One demonstrated that primacy effects were observed when memory was probed with a full report task. Experiment Two confirmed this finding while controlling eye movements. Critically, Experiment Three demonstrated that switching from a full to a partial report task abolished the primacy effect and produced a recency effect, consistent with the idea that the distribution of resources in VSWM depends on the type of recall required. It is argued that the primacy effect in the whole report task arose from the accumulation of noise caused by the execution of multiple spatially directed actions during recall, whereas the recency effect in the partial report task reflects the redistribution of preallocated resources when an anticipated item is not presented. These data show that it is possible to reconcile apparently contradictory findings within the resource theory of spatial working memory and the importance of considering how memory is probed when interpreting behavioural data through the lens of resource theories of spatial working memory.

## 5. Challenging the Bouma Law in Visual Crowding

Ramakrishna Chakravarthi, Amarachi Orisakwe and Nabeelah Young
School of Psychology, University of Aberdeen

**Abstract:** Visual crowding is the reduction in our ability to identify objects in clutter. The Bouma law posits that the range over which this interference occurs is a constant proportion (roughly 0.5) of the target’s eccentricity. However, this proposal of a ‘fixed’ zone of interference has been challenged by two strains of crowding studies. First, experiments that manipulated grouping between objects argue that the extent of interference depends on the organisation of the entire visual scene. Second, studies using dense arrays suggest that the nearest flankers and their similarity to the target are the primary determinants of crowding. Here, we examined these claims by manipulating density and grouping of flankers. We tested participants on an orientation discrimination task of a target presented at 6 deg eccentricity surrounded by dense ‘rings’ of flankers. In one experiment, we manipulated the number of flankers (4 or 124) and the similarity of the nearest flankers with the target. In sparse arrays, the effect of similarity depended on the flankers’ location, replicating the radial-tangential asymmetry in crowding. However, in dense arrays, the similarity of the nearest flankers barely modulated performance. In a second experiment, we found that, in dense displays, similar rings flanking the target led to more crowding than dissimilar ones. Interestingly, performance improved and reached a plateau with increasing number of innermost rings that were similar to each other. Taken together these results indicate that crowding extends beyond the nearest ring but is spatially constrained, whose extent depends on the organisation of the flankers.

## 6. An Introduction to the “Rocking Line” Illusion

Ian M. Thornton ^1^ and Dejan Todorović ^2^
^1^ Department of Cognitive Science, Faculty of Media & Knowledge Sciences, University of Malta, Msida, Malta
^2^ Laboratory of Experimental Psychology, Department of Psychology, University of Belgrade, Belgrade, Serbia

**Abstract:** In static displays, it is well known that the contrast between background and inducing elements can give rise to strong orientation illusions. The café wall illusion and Akiyoshi Kitaoka’s compelling effects are probably the best-known examples. Todorović (2021) provided an extensive review of such “polarity-dependent orientation illusions”, and used simulations to argue that they may arise due to the occurrence of “oblique clusters” in the population output of simple cells. Here, we present a new dynamic display to further explore such effects. In the “rocking line” illusion, a black inducing rectangle moves smoothly through the center of a series of horizontally staggered white rectangles that span the screen midline, forming a narrow checkboard pattern on a mid-gray background. When viewed at full size (all rectangles ≃ 2° VA), a veridical impression of horizontal motion occurs. However, when the display is säcaled down in size, a compelling impression that the inducing rectangle rocks around its own center as it moves, begins to dominate. The exact point at which the rocking appears, and the size at which it peaks, varies from observer to observer, but none fail to see it. The effect is probably closely related to the footstep/inchworm illusion (Anstis, 2001) and also possibly to the slalom illusion (Cesaro and Agostini, 1998). We believe the minimal nature of the display and its clear dependence on scale make it a particularly useful tool for exploring the spatial and temporal mechanisms that underly the perceptionof orientation in dynamic contexts.

## 7. Temporal Dynamics of Serial Dependence in Face Perception and Visual Working Memory

Anette Lidström
Department of Psychology, Lund University, Sweden

**Abstract:** Serial dependence (SD) refers to the influence of a recent stimulus on a person’s perceptual judgement of a current stimulus. SD has been found to arise for a wide variety of objects and features, including faces. Perceptual and mnemonic processes, along with response and decisional biases, are thought to contribute to SD effects. Here, two experimental studies are reported examining the time course of SD face effects, in attempt to determine the primary functional loci of such effects. In Experiment 1, participants were shown a series of two sequentially presented faces separated by an inter-stimulus interval (ISI) of 1, 3, 6, or 10 s and were instructed to match an adjustment face to the second face after a varying response delay of 0, 1, 3, 6, or 10 s. A statistically significant interaction was obtained between ISI and delay, where SD effects most consistently arose for ISI of 1 s and delays of 1 and 6 s. In Experiment 2 the ISI was held constant at 1 s and participants were post-cued to respond to either the first or the secondly presented face. For responses to the second face, SD effects again consistently arose with delays of 1 and 6 s, but not when responses were made to the first face. In all, the results suggest that SD face effects are separable from memory interactions and arise as a result of perceptual and mnemonic processes in a temporal fashion, and not purely as a result of response and decisional biases.

## 8. What Exactly Is So “Super” about Super-Recognisers?

Isabel M. Gillert ^1^, Claire Rogers ^1^, Josh P. Davis ^2^, Gnanathusharan Rajendran ^1^ and Louise S. Delicato ^1^
^1^ Herriot-Watt University, Edinburgh
^2^ University of Greenwich

**Abstract:** Super-recognisers (SR) are people with superior face recognition and identification abilities. We test whether this superiority extends to emotion perception by measuring their sensitivity to different facial expressions and comparing this sensitivity to other participants. This will help disentangle skills of face identity and emotion detection. Participants were grouped into SRs, Intermediate, and Controls depending on test-battery-scores. Sensitivity to emotions is determined by a task in which participants judged which of two successively presented faces was more expressive. One face was 0% expressive (neutral), and the other varied between 1–100% expressive. Sensitivity was measured as intensity needed to identify the more expressive face on 82% of trials. SRs and Intermediate participants were more sensitive to emotions than Controls. All participants were most sensitive to happy, followed by disgust, surprise, fear, anger, and least sensitive to sad expressions. Inverting the faces decreased sensitivity across all emotions with the largest inversion effects observed for sadness and anger. Visual characteristics that define sad and angry expressions are ambiguous and are often confused with other emotions, especially when inverted. This could account for the particularly high intensity needed to detect angry and sad expressions for upright and inverted faces.

## 9. Excessive Searching in Visual Search

Amelia R. Hunt ^1^, Anna Nowakowska ^1^, Stella Lin ^1^, Eden Reddy ^1^ and Alasdair D.F. Clarke ^2^
^1^ School of Psychology, University of Aberdeen
^2^ Department of Psychology, University of Essex

**Abstract:** An efficient decision about when to give up search and decide the target is absent depends on having an accurate representation of how easy it would be to find the target if it were present. We aimed to measure the strength of the relationship between target absent response times and several different estimates of target discriminability. Participants searched for a line segment oriented 45° to the right in a 22 × 16 grid of distractor lines. We manipulated discriminability by varying the heterogeneity of the orientation of the distractor line segments. In the first experiment, participants experienced blocks of unlimited and short (200 ms) duration search trials. To the extent that participants use discriminability of targets in deciding how long to search, the size of the drop in accuracy from long to short-duration trials should predict how much longer participants search before giving up in the long duration trials compared to the short, but there was no relationship between these measures. In a second experiment, we use a staircase to estimate the threshold duration needed to detect targets, and also asked participants to estimate how likely they would be to find targets in easy and hard displays. The results provide an estimate of what proportion of target absent response times can be attributed to actual, as well as expected, target discriminability. Our results suggest reaction times are generally longer than they need to be, and that a large proportion of variability in search performance is due to factors other than target discriminability.

## 10. Scarcity Effects (or Lack of) in Visual Foraging

Heather Statham, Anna Hughes and Alasdair Clarke
Department of Psychology, University of Essex

**Abstract:** Visual foraging is a task in which participants collect a sequence of targets from a display. Individuals adapt their foraging strategies depending on factors such as target value. During a trial, the relative number of each target type will vary based on previous selections, affecting how common each target is at any given point. While Brock’s Commodity Theory suggests individuals prefer a scarcer target due to its intrinsic value, it remains unclear to what extent this plays a role in foraging behaviour. Our Registered Report tests whether participants preferentially collect scarce targets based on an assumption of implicit value, and whether the Clarke et al. (2021) foraging model is able to account for the experimental data. Participants were asked to collect targets of two types, the ratios of which were manipulated to either make them equal or one scarcer than the other. Targets were polygons with seven or eight sides while distractors were polygons with six sides. An easy variant of the task had distinct colours for each of the targets and distractors, meaning that colour was informative. A hard variant of the task had the colours mixed so that colour was not informative. Participants each took part in one easy condition and one hard condition. Preliminary analysis of pilot data suggests that scarcity does not affect preference behaviour. This talk will present data from 36 participants that will be collected as part of the Registered Report.

## 11. Observers Can Adapt Their Search Strategy on a Trial-by-Trial Basis

Anna Nowakowska and Amelia Hunt
School of Psychology, University of Aberdeen

**Abstract:** Search strategies can vary considerably within participants when surface level properties of the stimulus are changed (Nowakowska et al., submitted). We asked participants to search for a line segment tilted 45 degrees to the right, or for a specific desktop icon. Critically, we divided the screen in two halves whereby the target was easy to find on one side of the screen centre and hard to find on the other side. Under such circumstances an optimal strategy is to concentrate search on the hard side; moving the eyes into the easy side provides no new information because the target can be detected on that side using peripheral vision. We found striking differences in performance between the conditions: When the target was an icon, participants used an optimal strategy, but when the target was a line, most participants fixated the easy side unnecessarily. We blocked and counterbalanced stimulus presentation in the original experiment as we were expecting participants would converge on one strategy if the trials were intermixed. In the current experiment we randomly interleaved the trials to test that prediction. Surprisingly, we found that most observers do flexibly shift from an optimal strategy when searching for an icon to variable (and counter optimal on average) strategy in case of lines on a trial-by-trial basis. The results suggest strategies are closely associated with stimulus properties, and that inefficient search of line segments is a stubborn behavioural tendency that cannot be remedied by intermixing a stimulus set that provokes efficient search.

## 12. The Influence of Target Identity on the Efficiency of Eye Movements during Visual Search

Manjiri Bhat ^1^, Anna Nowakowska ^1^, Amelia Hunt ^1^ and Alasdair Clarke ^2^
^1^ School of Psychology, University of Aberdeen
^2^ University of Essex

**Abstract:** Previous investigations of visual search efficiency have uncovered large individual differences that are stable over time yet highly sensitive to surface-level changes in the search context. In particular, search for objects based on their identity is much more efficient than search based on orientation. Across two experiments we investigated whether identity-based target templates can boost search efficiency even when the stimulus itself remains the same. In the first experiment, participants completed two blocks of search trials in which they indicated the presence/absence of a line tilted 45° to the right. In between the two blocks we induced an identity-based connection between the participant and search target using the self-reference effect (SRE). In this SRE-induction procedure, participants learnt to associate the target line to the label “you” and distractor lines to the label “stranger”. In the second experiment we used the same paradigm but altered the surface level property of the search array by embedding the line in a circle and adding two dots to create face pictograms. Overall, participants demonstrated a clear self-reference effect for the target and a general improvement in visual search in terms of accuracy post-SRE induction. This was, however, not reflected in any consistent change in the efficiency of participants’ search strategy. The results suggest changing a person’s connection to the target does not produce the robust effect on search strategies observed when the physical properties of the target itself are changed. Other interesting changes were observed, however, which will be presented as future directions for exploration.

## 13. Action Perception in Athletes Is Underpinned by Cue Use

Róisín Harrison, Martin Giesel and Constanze Hesse
School of Psychology, University of Aberdeen

**Abstract:** Prior research has demonstrated that athletes outperform non-athletes on action perception tasks involving anticipation of sport-related actions. We conducted two experiments to determine whether this advantage persists in tasks without anticipation and/or transfers to non-sport actions. In Experiment 1, motor experts (sprinters) and non-experts were shown two consecutive videos of an athlete either walking or sprinting. Participants’ task was to indicate whether the videos were identical or different. The sprinters were more accurate on the task for both sprint and walk actions, indicating that motor expertise can enhance perception of both expert and everyday actions. Further analysis revealed that participants who based their decisions on a specific cue (i.e., the distance between where the athlete’s foot landed and a line on the track) outperformed those who did not. However, the sprinters benefitted more from using this cue than the non-sprinters. Experiment 2 assessed whether non-experts’ performance could improve if the number of available cues was reduced and thus the informative cue could be identified more easily. Non-experts completed the same task as in Experiment 1, with half of the participants viewing the upper part of the athletes’ body and the other half viewing the lower part containing the informative cue. However, the non-experts still did not reliably identify the cue and the performance did not vary between the two groups. The results of these experiments suggest that motor expertise indirectly affects action perception by improving experts’ ability to identify and use informative cues.

## 14. Applying Bayesian Hierarchical Modelling to Assess the Speed of Goal Side Selection in a Soccer Related Penalty Shot Task

Geoffrey R. Patching
Department of Psychology, Lund University, Sweden

**Abstract:** Traditionally, psychophysical data is modelled by fitting individual curves to each participant’s data and then by statistical analysis of the extracted parameters. A now viable alternative to this traditional two-stage approach is Bayesian hierarchical modelling (BHM). BHM allows for modelling all of the data and all of the parameters in one-step rather than two. Here, BHM is used to analyse the speed of participants’ binary goal side selections in a soccer related penalty shot task. Participants viewed realistic images of a soccer goal and goalkeeper, and chose which side of the goal to best score. Analogous to the visual line bisection task, we (Pereira & Patching, 2021, Perceptual and Motor Skills, 128, 2279–2303) systematically repositioned the goalkeeper from left to right along the goal line and, to simulate changes in the viewing position of the kicker, lateral position of the goalmouth in each image. Scaling response times in terms of signed response speed shows a close linear correspondence with log odds ratios (logit) of left goal side selection. Overall, participants tended to choose the left- over right-goal side, but both the speed and direction of this tendency depended on the goalkeeper’s position and lateral position of the goalmouth relative to participants’ body midline. Current analysis of signed response speed shows a similar pattern of results as binary response probability complementing our earlier analysis of binary goal side selection in the present penalty shot task.

## 15. Effect of Subjective Visual Awareness on Multisensory Integration: Computational Model Analysis of Manual Reaction Times

Sanni Ahonen ^1^, Thomas Otto ^2^ and Arash Sahraie ^1^
^1^ University of Aberdeen
^2^ University of St Andrews

**Abstract:** In the redundant signal effect (RSE), reaction times to multiple simultaneously presented signals are faster than what can be expected based on RTs to the individual signals. Typically, participants are aware of the targets presented, however, the relationship between RSE and awareness is not clear. In this experiment, Continuous Flash Suppression (CFS) was used to reduce awareness of visual targets, which were presented alone or together with auditory targets. Twenty-four participants’ manual RTs were recorded alongside subjective awareness of visual targets. Reaction times were faster in the multisensory condition only when participants reported having subjective awareness of the visual target. We used the Shifted Wald (SW) model to further investigate the interactions between subjective awareness and multisensory processing. The model parameters differed between multisensory trials where participants reported being aware of the visual targets and multisensory trials without awareness of the visual target. A within-subjects comparison of standardised model parameters showed that, for each participant, the parameter reflecting non-decision time (θ) was lower when they were aware of the visual target compared to when they lacked awareness. The stimulus strength was identical in each trial, suggesting the differences were brought on by trial-by-trial fluctuations in neuronal noise. Our findings support the notion that dynamic neuronal noise influences the probability of a sub-threshold visual target entering conscious awareness. We suggest that the relationship between subjective visual awareness and neuronal noise is reflected in the θ parameter. These findings provide further evidence that subjective visual awareness affects multisensory RSE.

## 16. Adaptive Estimation of Continuous Object Position

Justin Ales
School of Psychology and Neuroscience, University of St Andrews

**Abstract:** Most psychology experiments use a rigid trial-based structure. This structure ignores the fact that in the real-world things rarely follow this discrete and well-separated structured. To understand real-world behaviour methodologies that enable probing and understanding behaviour in real-time will enable us to move beyond this rigid trial structure. This experiment utilized a paradigm in which participants behaviour (pointer location) was continuously tracked while they indicated the position of a continuously moving object. On top of the dynamic stimuli step function changes in stimulus location were introduced as probes. Step-function inputs are a commonly used in control-systems to study system behaviour they provide rich sources of information for the dynamics of behavioural responses. By embedding step inputs in different contexts we can therefore determine whether and how humans dynamically adjust their estimation strategy. In this study we adjusted stimulus factors that affect how reliably a participant can estimate the stimulus location. In response to changes in stimulus reliability participants changed the dynamics of their tracking behaviour. This supports the theory that people use adaptive information integration strategies that dynamically adjusts in response to stimulus reliability.

## 17. Virtual Reality for Vision Science: New Stimuli and New Ways of Seeing

Paul Hibbard
Department of Psychology, University of Essex

**Abstract:** Virtual reality provides an immersive and responsive perceptual experience. It allows us to simulate the ambient optical array of a moving observer with full and replicable control of the environment, while providing accurate ground truth information about the structure, location and identity of objects and surfaces. This provides many opportunities for the study of visual processing using naturalistic stimuli and tasks. These new stimuli allow us to study vision as a way of seeing in a 3D environment, rather than the study of 2D patterns or pictorial space. By considering both the visual stimuli we present to our observers, and the tasks that we set them, we can define a continuum of ways of seeing. At one end, traditional psychophysics addresses our ability to detect and discriminate between visual patterns. If these patterns are interpreted as objects and surfaces, then gauge figure and other tasks can be used to measure the properties of pictorial space. The inclusion of accurate binocular and motion parallax cues provides an intermediate experience, with a precisely defined point of view into this pictorial space. Finally virtual reality allows us to study perception in the 3D space inhabited by the observer. The creation of virtual environments using games engines, and their presentation using virtual reality, allows us to avoid many of the confusions that arise when our experiments are constrained within pictorial space.

## 18. How Do Bumblebees Approach Objects When They Are Iridescent?

Li Shiwen, Alexandra E. Reynard, Natalie Hempel de Ibarra, Rebekah C. White and Hannah E. Smithson
Department of Experimental Psychology, University of Oxford, Woodstock Road, Oxford OX2 6GG, UK

**Abstract:** Recent empirical evidence has shown that some animals discriminate between iridescent and non-iridescent surfaces, and moreover, that iridescence leads to measurable changes in foraging behaviour. For iridescent surfaces the colour of light reflected from the object is determined by the geometric relationship between the object, light source, and viewing angle of the observer. An open question remains as to which visual features allow the observer to distinguish iridescent from non-iridescent surfaces, and whether observers—such as bees—will actively adjust their behaviour to sample those visual features. This is particularly interesting in the context of bees inspecting flowers as some species of flowers have physical surface properties that produce iridescent effects. Thus, depending on flight manoeuvres and viewing positions adopted by a bee, the bee might be able to gain access to different signals (photoreceptor values) from the same flower for their foraging or navigational decisions. We present an exploratory study of the flight behaviour of bumblebees (Bombus terrestris). During the experiments, the bees had access to a flight arena, in which a sugar syrup was offered near to a stimulus which was ~10 × 19 mm opaque blue glass coated in translucent film (i.e., non-iridescent) or iridescent film (i.e., iridescent). We manipulated the geometric relationship between the stimulus and light source and measured the bees’ body angles, approach angles, hovering, and landing locations. Early data (15 landings per stimulus orientation and coating) suggest behavioural differences for iridescent and non-iridescent samples.

## 19. A Dynamical Firing Rate Model of Optic Flow Integration in the Sky Compass Network in the Brain of the Desert Locust

Kathrin Pabst ^1^, Uwe Homberg ^2^ and Dominik Endres ^1^
^1^ Department of Psychology, Philipps Universität Marburg, Germany
^2^ Department of Biology, Philipps Universität Marburg, Germany

**Abstract:** The central complex (CX) is the navigation hub of the insect brain. Evidence from the desert locust Schistocerca gregaria suggests that it houses a 360° representation of heading direction relative to sky compass cues (Pegel et al., 2019 J. Neurosci, Zittrell et al., 2020 Proc. Natl. Acad. Sci.). A robust representation is most likely achieved by integrating other cue modalities, such as rotational optic flow, which certain CX neurons respond to (Zittrell et al., 2022 bioRxiv). We implemented a dynamical firing rate model of CL1a compass neurons and rotation-sensitive CL2 neurons constrained by the aforementioned physiological and available anatomical data (Heinze and Homberg 2008 J. Comp. Neurol.). We tested whether synaptic weights could be optimized such that the activity profile of the network would remain stable over time or shift in tune with angular velocity information conveyed by two TN neurons putatively providing optic flow signals. The network can maintain a sTable 360° compass signal that is shifted via rotation-dependent asymmetrical synaptic modulation. During a simulated movement trajectory, the network compass signal aligns with the ground truth bump position, also in the presence of noise. Insect navigation is a productive model behavior, and future research will reveal if similar processing principles also apply in other species, e.g., primates.

## 20. Feature-Based Attention during Smooth Pursuit Eye Movements

Nika Adamian ^1^, Benjamin W. Tatler ^1^ and Søren K. Andersen ^1,2^
^1^ School of Psychology, University of Aberdeen, UK
^2^ Institute for Psychology, University of Southern Denmark, Denmark

**Abstract:** We use eye movements to direct attention to the behaviourally relevant parts of the visual field. At the same time, we can attend to simple features such as colour or shape. Feature-based attention has traditionally been studied in the absence of eye movements, leaving open the possibility that active vision shapes feature selection. To test this, we compared the magnitude of feature-based attention during central fixation and smooth pursuit eye movements. Participants (n = 21) observed two spatially overlapped circular fields of randomly moving red and blue dots. The aperture containing this target stimulus was presented against a full-screen background also filled with spatially overlapped red and blue dots. On each trial, participants were cued to attend to one of the two colours within the task aperture and detected brief luminance decrements in the attended colour. In separate blocks of trials, the target aperture was either static (Fixation condition) or followed a circular path around the screen at a constant velocity (Smooth pursuit condition). Steady-state visual evoked potentials (SSVEPs) elicited by each part of the stimulation (red and blue dots within and outside the task aperture) were used to measure the magnitude and global spread of feature-based attention. The results showed that although there was a behavioural cost associated with deploying eye movements, the magnitude of attentional selection did not differ between conditions, both within and outside the spatial focus of attention. This suggests that feature-based attention does not interact with smooth pursuit eye movements at the level of early visual cortex.

## 21. Frontal Visual Response Fields Integrate Target and Landmark Cues for Gaze Control

J. Douglas Crawford ^1,2^, Vishal Bharmauria ^1,2^, Adrian Schütz ^2,3^ and Frank Bremmer ^2,3^
^1^ Centre for Vision Research and Vision: Science to Applications Program, York University
^2^ Brain in Action International Research Training Program
^3^ Department of Neurophysics, and Center for Mind, Brain and Behavior, Philipps-Universität Marburg

**Abstract:** The visual system has access to two cues for spatial behavior: egocentric (relative to the self) and allocentric (relative to the external world). This distinction is important in the dorsal (egocentric) and ventral (allocentric) visual streams (Milner and Goodale 2006; Schenk 2012). Recently, behavioral, neuroimaging and neurophysiology studies suggest that these cues are then optimally integrated for goal-directed action (Byrne & Crawford 2010; Chen and Crawford 2020; Bharmauria et al., 2020, 2021). However, it remains unclear how these cues are multiplexed for input to the motor system. To investigate this, we trained monkeys to direct gaze toward remembered visual targets in the presence of a large visual landmark, while recording from the frontal (FEF) and supplementary (SEF) eye fields. To determine spatial codes in these areas, we fit visual response fields (to the target) against various spatial models, including target/landmark coding in egocentric, allocentric, and intermediate frames of reference. Most FEF response fields preferentially coded target location relative to the eye, but a substantial minority (30%) encoded landmark location. Further, cells that coded both targets and landmarks also showed a shift in target coding toward an intermediate target-landmark reference frame. Similar results were found in the SEF. Overall, these results show that prefrontal cortex retains and integrates visual landmark signals with eye-centered target signals, providing the necessary information for optimal integration in the gaze system. This could be a general sensorimotor mechanism, allowing integrated ego/allocentric visual cues to provide the most reliable estimate of target location.

## 22. Binocular Portraits

Nicholas J. Wade
Psychology, University of Dundee, Dundee DD1 4HN, UK

**Abstract:** Stereoscopy and photography were announced to the public at about the same time: stereoscopy (by Wheatstone) in 1838 and photography (by Daguerre and Talbot) in 1839. Wheatstone enlisted Talbot to take the first stereoscopic photographs (of a statue) and many stereoscopic portraits were made thereafter. Visual scientists, particularly in Germany, were more cautious about embracing stereoscopy because the experience of depth from stimulation of non-corresponding retinal points undermined the prevailing theory of binocular single vision. This early history of the application and theory of vision with two eyes is presented with anaglyphic portraits of the scientists and photographers involved, like Wheatstone, Brewster, Talbot and Claudet. The introduction of random dot stereograms by Julesz a century later has extended the possibilities for creating binocular portraits. In addition, the development of reflecting and refracting stereoscopes is illustrated with anaglyphs: Wheatstone’s mirror stereoscope was made in London in 1832 whereas Brewster’s lenticular (refracting) stereoscope was made in Dundee in 1849. Keywords: anaglyphs, portraits, photography, stereoscopic vision

## 23. The Effects of Naturalistic Backgrounds and Movements on Judgements of Motion

Hollie Carter, Julie Harris and Justin Ales
School of Psychology and Neuroscience, University of St Andrews

**Abstract:** Predators must distinguish the movements of their prey from their environment and other animals. Therefore, camouflaging one’s motion, in static and dynamic environments is valuable. Several motion camouflage strategies have been suggested to help prey evade predators. However, the effectiveness of these strategies has not been quantified. Our aim was to measure if the strategies are effective in an environment emulating natural motions. We started by creating a 3D-modelled database of moving forest scenes and prey movements. The database consists of variable landscapes, vegetation, composition, lighting, wind speeds, materials, scale, and viewing distances. Next, we measured human performance by asking participants to click a location to indicate perceived prey motion direction, in short video clips of the moving forest scenes. A small prey target moved in one of four motion patterns, inspired by motion camouflage strategies: (1) straight; (2) punctuated—prey stops and starts on a straight path to limit the predictability of motion; (3) sinusoidal; (4) zigzag—emulating ‘protean motion’ proposed to hinder location estimation. These were tested over 8 orientations and 2 environments (plain grey and moving forest). Both environment and motion type impacted motion estimation. Dynamic environments resulted in more variance in direction estimation than plain environments, across all motion types and orientations. Sinusoidal and zigzag motion produced greater systematic error and variance than straight and punctuated motion. Our work has begun to quantify how prey might have evolved motion to counter the exquisite motion detection abilities of many predator visual systems.

## 24. Some Observations about Binocular Motion and Depth Processing

Martin Lages
School of Psychology and Neuroscience, University of Glasgow

**Abstract:** The predominant idea of binocular motion and depth processing is to establish pointwise correspondences between images in the two eyes in order to resolve the correspondence problem of motion and depth. If local edge detectors are the front end of motion and depth encoding, then the corresponding motion and depth constraints from the left and right eye are insufficient to infer the 3D motion direction of a contour inside a small receptive field. Although additional features such as corners, T-junctions and intersections can disambiguate 3D motion this would require specialised receptive fields that can accommodate different orientations of corresponding features in the left and right eye. Maintaining binocular receptive fields for all possible combinations of spatial and temporal characteristics in the two eyes seems costly and inefficient. Instead, it is more likely that separate pathways for binocular motion and depth processing interact with abstractions of global 3D object motion. Combining appropriate priors with motion and depth constraints of an object moving in 3D may provide a more effective way to approximate binocular 3D motion. This would enable the visual system to process detailed spatial information while using relatively coarse motion information. In examples, advantages and limitations of this general approach are highlighted.

## 25. The Stereoscopic Anisotropy Is Present in Frontoparallel Cyclopean Motion Using Suprathreshold Disparities

Ichasus Llamas-Cornejo ^1^ and Ignacio Serrano-Pedraza ^1,2^
^1^ Department of Experimental Psychology, Universidad Complutense de Madrid, 28223 Madrid, Spain
^2^ Centre for Behaviour and Evolution, Newcastle University, Newcastle upon Tyne NE2 4HH, UK

**Abstract:** We investigated cyclopean (stereoscopic) motion in the frontoparallel plane measuring duration thresholds for different stimulus parameters: disparity, spatial frequency, temporal frequency, and stimulus size. To construct cyclopean motion, we used sinusoidal disparity corrugations obtained with random dots stereograms that changed every frame at 120 Hz for each eye. Using Bayesian adaptive-staircases, we measured duration thresholds for detecting the correct direction of motion of horizontal (left-right) and vertical (up-down) corrugations. In the first experiment, we tested the effect of disparity for 0.4 c/deg drifting at 2 Hz. Duration thresholds decreased with increasing disparity until 2.3–2.5 log10(arcsec) then, thresholds remained constant. The other experiments were performed with disparities between 2.4–2.5 log10(arcsec). In the second experiment, we tested the effect of size (from 8 to 22 deg) for two spatial frequencies, 0.2 and 0.4 c/deg drifting at 2 Hz. Results show that duration thresholds decreased with increasing size until 8–16 deg, then duration thresholds remained constant. In the third experiment, we tested different temporal frequencies (range 0.5–4 Hz) for vertical and horizontal sinusoidal corrugations of 0.2 c/deg. Results show that duration thresholds decreased with increasing temporal frequency until 1–2 Hz and then remained constant. From 2 to 4 Hz, a strong anisotropy was present, where vertical corrugations showed higher duration thresholds than horizontal corrugations. Our results show, for the first time, a strong stereomotion anisotropy using suprathreshold disparities. Our results also show no surround suppression in stereomotion and also, lower duration thresholds when increasing disparity, that is the opposite result to increasing contrast in the luminance domain.

## 26. Induced Motion at Short Durations Reveals a Strong Interaction between High and Low Spatial Frequencies

Omar Bachtoula ^1^ and Ignacio Serrano-Pedraza ^1,2^
^1^ Department of Experimental Psychology, Universidad Complutense de Madrid, 28223 Madrid, Spain
^2^ Centre for Behaviour and Evolution, Newcastle University, Newcastle upon Tyne NE2 4HH, UK

**Abstract:** Induced motion occurs when a static stimulus seems to drift in the opposite direction of a moving surround. The aim of this study was to characterize this illusory motion, using different spatial and temporal frequencies, viewing conditions (binocular and dichoptic), and short stimulus presentations. We conducted two experiments, where the stimuli contained a vertical grating of 1 c/deg (test) surrounded by a circular band with another vertical grating (inducer). Using a Bayesian adaptive procedure, we measured the intensity of motion induction by means of the speed of the test that cancelled the illusory motion produced by the inducer’s drift (i.e., cancellation speed). In the first experiment, we determined the effect of the surround’s spatial frequency (0.5–6 c/deg) on the cancellation speed for two brief stimulus durations (50 and 100 ms). Results showed a band-pass tuning of the cancellation speed as a function of the surround’s spatial frequency, with the peak at 2 c/deg for the shortest duration. In the second experiment, we estimated the influence of the surround’s temporal frequency (2–24 Hz) on the cancellation speed at short durations (50 ms). The cancellation speed increased as temporal frequency increased up to 12–16 Hz, from which it remained constant. Overall, binocular presentations induced a stronger illusory motion on the central component than dichoptic viewing, and the shortest duration increased the intensity of motion induction. These results reveal a strong interaction in motion perception between high and low spatial frequencies when test and inducer are presented briefly in different retinal positions.

## 27. Characterizing Reversals in Motion Discrimination When High and Low Spatial Frequencies Are Combined

Sandra Arranz-Paraíso ^1^ and Ignacio Serrano-Pedraza ^1,2^
^1^ Faculty of Psychology, Complutense University of Madrid, 28223 Madrid, Spain
^2^ Institute of Neuroscience, Newcastle University, Newcastle upon Tyne NE2 4HH, UK

**Abstract:** Motion direction discrimination of drifting high spatial frequency pattern becomes impaired when a static low spatial frequency pattern is added to it (Derrington & Henning, 1987; Serrano-Pedraza, Goddard & Derrington, 2007). It has been suggested that the impairment is due to an inhibitory interaction between motion sensors tuned to high and low spatial frequencies (Serrano-Pedraza et al., 2007). Although it is known that the strength of the interaction is higher at short stimulus durations (between 25 and 100 ms), up to now it has not been studied for very short durations (<25 ms). In this study, using a PROPixx projector at 1440 Hz, we tested nine durations: 2, 5, 10, 20, 30, 40, 60, 80, and 120 ms. Using a forced-choice motion discrimination task, the proportion of correct responses was obtained through the method of constant stimuli. We tested vertical Gabor patches of 46% of contrast, two speeds of 2 and 4 deg/s, and two sizes 4 and 8 deg diameter. Five stimuli were tested: complex stimuli, 3 c/deg static added to a 1 c/deg moving (3 s + 1 m), 3 c/deg moving added to a 1 c/deg static (3 m + 1 s), 3 c/deg moving added to a 1 c/deg moving (3 m + 1 m), and simple stimuli 3 c/deg moving and 1 c/deg moving. We only found reversals in motion discrimination in the 3 m + 1 s condition for durations between 20 and 40 msec and a size of 4 deg. Thus, reversals are present within a small range of short durations and are size dependent. [Supported by Grant No. PDI2021-122245NB-I00 to ISP from Ministerio de Ciencia e Innovación, Spain].

## 28. Summation and Differencing Channel Adaptation Affects Motion-in-Depth Speed Discrimination

Lauren Murray
Department of Psychology, Faculty of Natural Sciences, University of Stirling

**Abstract:** The efficient coding principal provides an alternative approach to understanding binocular coding, where disparity-sensitive neurons respond to the sum and difference of left and right eye inputs, reducing signal redundancy. To explore the theory’s potential role in motion-in-depth perception, we measured speed-in-depth discrimination, following the independent adaptation of the two channels. We recovered psychometric functions for discrimination performance at three standard test speeds (27, 53 and 107 mm/s) following the presentation of two adaptors: correlated adaptors used to adapt the summation channel, and anticorrelated to adapt the differencing. Both 1/f filtered noise patterns and 1D horizontal noise adaptors were used in separate blocks. Perceived speed was expected to reduce following differencing channel adaptation and increase following summation channel adaptation. Adaptation and test stimuli were presented above and below fixation, with each adaptor type presented in only one location per block, counterbalanced across blocks. Participants selected which of the two test stimuli moved fastest. Points of subjective equality (PSEs) were measured for intervals where the standard speed-in-depth stimuli were presented in the same location as the correlated or anticorrelated adaptors. As anticipated, PSEs indicated a reduction in perceived speed at the anticorrelated adaptor location, and an increase at the correlated location. Also, adaptation effects were reduced at faster speeds, suggesting the involvement of a second binocular mechanism: interocular velocity differences (IOVD). In line with previous findings, we also found an improvement in discrimination performance with increasing speed, consistent with speed-in-depth perception depending on contributions from both CD and IOVD cues.

## 29. Increasing Parallactic Change Compresses Depth and Perceived Distance

Xue Teng, Laurie M. Wilcox and Robert S. Allison
Centre for Vision Research, York University, Toronto, Canada

**Abstract:** Motion parallax provides information for both absolute distance and relative depth judgments. For a given head motion and given depth interval, the parallactic change is inversely proportional to the square of egocentric distance. In this presentation we will discuss analysis of a subset of data from a larger study. On each trial, monocularly-viewing observers made left-right swaying head motions at 1.0 Hz to induce the corresponding virtual motion shown on a head mounted display. A gain distortion was applied to the virtual motion, ranging from half to twice the physical motion. While moving, observers adjusted the angle of a vertical fold stimulus presented at distances from 1.3 to 6.0 m so it appeared to be at 90 deg. After the adjustment was made another virtual environment was presented. While standing stationary observers matched a pole to the apparent distance of the peak of the previously seen fold. On average observers adjusted the folds to have smaller depth as gain increased or distance decreased. Estimates of target distance also declined with increasing gain. As both distance and gain affect the amount of parallactic change we analysed to what extent our results could be explained by this variable alone. Our analysis confirmed that both of these measures varied consistently with parallactic change. We will discuss the implications of these findings for depth cue scaling, and for anticipated tolerance to tracking errors in virtual reality systems.

## 30. Measuring Variability in EEG Waveforms: Lessons from Visual Evoked Potentials

Jasna Martinovic
School of Philosophy, Psychology and Language Sciences, University of Edinburgh

**Abstract:** At the core of the event-related potential (ERP) method lies an assumption that observed ERPs reflect a manifestation of underlying latent ERP components, which are assumed to be either positive (e.g., P1) or negative (N1) in amplitude. ERP components may therefore be subject to rules that govern zero-bound measures. This poses a fundamental problem for ways in which we quantify variability of EEG waveforms, as in zero-bound measures those participants with higher mean responses also have more room for variability to manifest itself. To evaluate if this holds, I median-split an EEG dataset with 38 participants (Martinovic et al., 2018 Journal of Vision 18 4) into two subgroups based on the amplitude of their early sensory component P1. Higher P1 amplitude reflects more phase-locking to the stimulus and thus higher signal strength. I compare standard errors of the mean (SEMs) and coefficients of variation (CVs; standard deviation divided by the mean) between the low-P1 and high-P1 groups, finding that the high-P1 group has significantly elevated SEM throughout the trial i.e., including the baseline that precedes stimulus onset and reflects non-evoked neural oscillations. Unlike SEM, CV does not identify between-group differences in variability during baseline. In conclusion, to be suitable for quantifying data noisiness, a standardised ERP variability measure should also account for the mean.

## 31. Chromatic and Spatial Image Statistics Predict Infants’ Visual Preferences

Philip McAdams ^1^, Sara Svobodova ^1^, Kezia Terry ^1^, Taysa-Ja Newman ^1^, Megan Chambers ^1^, Jenny Bosten ^2^, Alice Skelton ^1^ and Anna Franklin ^1^
^1^ The Sussex Colour Group & Baby Lab, The School of Psychology, University of Sussex, Brighton BN1 9RH, UK
^2^ Sussex Vision Lab, The School of Psychology, University of Sussex, Brighton BN1 9RH, UK

**Abstract:** Natural scenes and art contain statistical regularities in features such as colour and space (Graham, & Redies, 2010). Adult visual perception and visual aesthetics appear to be tuned to these image statistics (Simoncelli & Olshausen, 2001). Of interest, is how visual perception tunes to image statistics during development, and what role this plays in the development of visual aesthetics. It has been suggested that infants’ visual preferences can reveal perceptual primitives of adult aesthetics (Göksun et al., 2014), e.g., infants look longer at colours that adults prefer (Skelton & Franklin, 2020). Here, we present a series of infant studies that investigate infants’ and adults’ visual preferences for natural scenes, art, and fractals. Do infants look longer at the stimuli that adults prefer, and is infant looking and adult liking driven by image statistics? We find that some aspects of adult aesthetic preference can be traced back to infants’ visual preferences. For example, infants look longer at the edge co-occurrence statistics found in natural scenes and that adults like; and that the amount of variation in the luminance and saturation of artworks contributes to infants’ visual preferences and adults’ aesthetic preferences. We also find that infants have a visual preference for fractals, that differs from adults’ preference, possibly suggesting an adapting role of visual experience on aesthetics; and that a combination of chromatic and spatial image statistics predicts infant looking to artworks. These studies potentially identify ‘perceptual primitives’ of aesthetics that can be traced back to early sensory biases in infancy.

## 32. Human Cortical Tuning for Binocular Disparity Reveals Discrete Classes of Neural Activations

Andrew J. Parker ^1,2^, Ivan Alvarez ^3,4^, Alessandro Mancari ^5^, I. Betina Ip ^3,4^ and Holly Bridge ^3,4^
^1^ Department of Physiology, Anatomy and Genetics, University of Oxford, Oxford, UK
^2^ Department of Sensory Physiology, Institute of Biology, Otto von Guericke University, Magdeburg, Germany
^3^ Oxford Centre for Functional MRI of the Brain (FMRIB), Wellcome Centre for Integrative Neuroimaging, University of Oxford, Oxford, UK
^4^ Nuffield Department of Clinical Neurosciences, University of Oxford, Oxford, UK
^5^ Department of Biological Sciences, Scuola Normale Superiore, Pisa, Italy

**Abstract:** Visual perception of fine depth is strongly dependent on binocular stereopsis, the ability to decode depth from the horizontal offset between the retinal images in the two eyes. While multiple cortical areas are associated with stereoscopic processing, it is unclear how tuning to specific binocular disparities is organized across human visual cortex. In this study, we examined fMRI responses (2 mm isotropic) to parametric modulation of binocular disparity to characterize the neural tuning to disparity across multiple visual areas. Participants observed a random-dot stereogram stimulus with varying disparity content, and responses were modelled using the haemodynamic response function to deliver a 1-dimensional tuning curve along the depth dimension for each sample point across the cortical surface. Disparity preference is not uniformly distributed, with an over-representation of near disparities in dorsal visual areas. Second, our data reveal the expected relationship between preferred disparity and tuning curve width, with sharply tuned disparity responses at near-zero disparities, and wider disparity tuning profiles encoding large near or far disparities. Third, principal components analysis and clustering analysis indicate distinct populations of tuning types, revealing selective tuning for near and far disparities from early visual areas (V1, V2) out to higher visual areas (LOC). These findings point to heterogeneous processing of disparity across human visual areas, suggesting that neurons sensitive to binocular stereopsis play different roles in different visual areas.

## 33. Can Cognitive Tests Differentiate Progressive Supranuclear Palsy from Parkinson’s Disease?

Alexis Cheviet, Alison Lane, Anthony Atkinson and Daniel T. Smith
Department of Psychology, Durham University

**Abstract:** Progressive Supranuclear Palsy (PSP) is a neurodegenerative disease characterized by a wide range of symptoms including falls proneness, mobility ifficulties, akinesia, axial rigidity, and vertical paralysis of the gaze. Owing to the large overlapping of these clinical signs with those reported in the idiopathic Parkinson disease (PD), PSP is often mistaken as PD, at least during the early stages of the pathology. Existing research suggests that people with PSP have problems with visuospatial attention and short-term memory as compared to PD patients, but these factors are not routinely used during diagnosis. The present tudy aims to test the relevance of these tests in patients suffering from PSP and PD as compared to age-matched control (AMC). Visual attention was assessed using three visual search tasks in which participant had to identify a target among distractors (defined by its colour, its orientation, or a conjunction of both). The short-term memory task required the subject to recall the colour or the position of one among several objects. Additionally, we used an emotion recognition task to assess social cognition and three tests (saccades, smooth pursuit and reading) to explore the oculomotor system integrity. Overall, PSP group performed worse in all tasks as compared to PD and AMC groups. Especially, visual search discriminated extremely well between PSP and PD patients in the orientation condition, potentially paving the way towards a new and cheap diagnostic tool for clinicians.

## 34. Colour Vision Screening Test Sensitivity before and One Week after Cataract

Zane Jansone-Langina and Maris Ozolinsh
Department of Optometry and Vision Science, University of Latvia, LV-1004 Riga, Latvia

**Abstract:** Introduction: In the current literature, there have not been attempts to determine colour vision sensitivity shift with classical screening tests like unsaturated Farnsworth D15 test and Hardy Rand and Rittler. Method: This study analysed 54 patients with senilis cataract (mean age 62.5 ± 0.7 years). The surgery was performed for both eyes with a two day delay between surgery of each eye. As a colour vision test we used unsaturated Farnsworth D15 panel test and Hardy Rand and Rittler (HRR) pseudoisochromatic plate tests. It comprises a 6 plate screening section plus 14 detailed diagnostic plates to determine the type and extent of colour deficiency. Results: Before the surgery 6 patients showed colour sensitivity shift to the tritan confusion axis side, did not show any colour sensitivity abnormalities after one week. Four eyes which showed normal cap arrangement sequence before the surgery, after showed nonspecific colour sensitivity changed closer to protan confusion axis. Before and after cataract surgery HRR test showed no colour vision deficiency problems. Conclusion: Before and 2 weeks after cataract surgery patient’s colour vision sensitivity changes. Using unsaturated Farnsworth D15 it is possible to detect colour sensitivity shift, which cannot be done with Hardy Rand and Rittler test.

## 35. Computerised Colour Vision Test

Renārs Trukša, Zane Jansone-Langina, Sergejs Fomins and Jānis Dzenis
Optometry and Vision Science Department, University of Latvia, Riga, Latvia

**Abstract:** It is recommended that at least two different tests be used to properly assess colour vision. Although the ability to resolve colour stimuli is crucial in modern society, for example, in education so that children with colour vision deficiencies do not lag behind their peers, or in the transportation industry so that lorry and train drivers and aeroplane pilots can correctly recognise signal lights and act accordingly to avoid accidents. A few decades ago, a new type of colour vision test emerged—computerised colour vision tests, two prominent examples being CCT and CAD. It has been shown that it is possible to use computerised colour vision tests to detect colour vision deficiencies and assess colour vision for different age groups. Research suggests that computerised colour vision tests would allow colour vision to be assessed with only one test. However, computerised colour vision tests require high-quality, colour-calibrated monitors. As part of our research, we developed a computer-based colour vision test that can be operated on consumer-grade colour-calibrated displays. Our test stimuli have two modifications, one similar to the CCT design and the other similar to the CAD test design. Chromatic sensitivity with both test stimuli was evaluated in 10 directions of the colour space. To evaluate the sensitivity and specificity of our version of the computerised colour test, participants’ colour vision was assessed with colour vision tests—HRR, FM—100, Oculus HMC, and CAD.

## 36. The Effect of Stimulus Contrast and Direction on Saccadic Eye Movement Parameters

Viktorija Goliskina, Ilze Ceple, Renars Truksa, Sergejs Fomins, Gatis Ikaunieks, Aiga Svede, Evita Serpa, Liva Volberga, Linda Krauze, Evita Kassaliete, Sofija Vasiljeva and Gunta Krumina
Department of Optometry and Vision Science, Univesity of Latvia

**Abstract:** Saccadic eye movement analysis as an objective measurement of the perceptual processes and has been extensively applied in various scientific fields. However, most of the studies analysing saccadic performance have applied different experimental setup (stimulus size, contrast, location, etc.). The current study aims to explore the effect of stimulus contrast and direction on saccadic eye movement latency, peak velocity, and accuracy measurements. Saccadic eye movement stimuli of different contrast levels were demonstrated at four different directions; eye movements were recorded with Tobii Pro Fusion video-oculograph (250 Hz). The results demonstrate a significant effect of stimulus direction on the latency and peak velocity measurements at medium grey background 30 cd/m^2^ (negative and positive stimulus polarity); light grey background 90 cd/m^2^ (negative polarity) and black background 3 cd/m^2^ (positive polarity). A significant effect of stimulus direction was observed on the accuracy measurements when the saccadic eye movement stimuli were demonstrated on medium grey background (negative polarity) and on black background. No significant effect of stimulus contrast was observed on peak velocity measurements under any conditions. Significant stimulus contrast effect on latency and accuracy was observed only on light grey background. Overall, it can be concluded that the best saccadic eye movement performance (lowest latency, highest peak velocity, and accuracy measurements) can be observed when saccades are oriented to the right and left from the central fixation point. Furthermore, when presenting the stimulus on a light grey background, a very low contrast stimuli should be considered carefully. Acknowledgement: This work has been supported by the Latvian Council of Science (project Nr.lzp-2021/1-0219), University of Latvia (project Nr. Y5-AZ77-ZF-N-100) and SIA Mikrotikls and University of Latvia Foundation (project Nr. 2260).

